# The Effects of Religiosity on Depression Trajectories Before and After Widowhood

**DOI:** 10.1093/geroni/igab046.2189

**Published:** 2021-12-17

**Authors:** Frances Hawes, Jane Tavares, Corina Ronneberg, Edward Miller

**Affiliations:** 1 University of Wisconsin-Eau Claire, eau claire, Wisconsin, United States; 2 LeadingAge LTSS Center @UMass Boston, Boston, Massachusetts, United States; 3 University of Illinois Chicago, Chicago, Illinois, United States; 4 University of Massachusetts Boston, Boston, Massachusetts, United States

## Abstract

Widowhood is associated with decreased emotional well-being, particularly increased depression. Religiosity may help improve mental health among widowed individuals. However, longitudinal studies exploring the role of religiosity on emotional well-being among widowed older adults is lacking, as are studies which examine this relationship using different dimensions of religiosity. This study analyzed data from the 2006-2016 waves of the nationally representative Health and Retirement Study (HRS). Trajectories of depression among older adults >50 years (N=5,486) were examined to explore patterns of depression among those entering widowhood and the potential impact of religiosity on depressive symptoms during widowhood. Ordinary least squares (OLS) regression analysis was used to examine the association between widowhood and depression as well as the role of religiosity as a moderator of this association. Older adults experienced an increase in depressive symptomology after the onset of widowhood, and although the levels of depressive symptomology decrease post-widowhood, they do not return to their pre-widowhood levels. Additionally, high religious service attendance and higher intrinsic religiosity were both associated with lower depressive symptomology. High religious service attendance moderated the relationship between widowhood and depression. The relationship between high religious service attendance and depression was stronger among widowed older adults living alone. This study highlights the long-term effects of widowhood on depressive symptomology among older adults. The findings also suggest that higher religious service attendance can lessen the effects of widowhood on depressive symptoms, especially for those living alone. These findings may inform intervention development around increased screening and treatment for depression.

